# Correlation between Cognitive Impairment and Functional Disability in Elderly People After COVID‐19

**DOI:** 10.1002/alz70857_107380

**Published:** 2025-12-26

**Authors:** Fabiana Carla Matos da Cunha Cintra, Marco Túlio Cintra, Gerson Santos Azevedo, João Vitor de Queiroz Campos, Gabriel Brant Moreira Ferreira, Júlia Dias, Iza de Faria Fortini, Bernardo de Mattos Viana, Maria Aparecida Camargos Bicalho

**Affiliations:** ^1^ Federal University of Minas Gerais, Belo Horizonte, Minas Gerais, Brazil; ^2^ UFMG, Belo Horizonte, Minas Gerais, Brazil; ^3^ Elderly Psychiatry and Psychology Extension Program (PROEPSI), Faculdade de Medicina, Universidade Federal de Minas Gerais, Belo Horizonte, Brazil; ^4^ National Institute of Science and Technology Neurotec R (INCT‐MM), Faculdade de Medicina, Universidade Federal de Minas Gerais, Belo Horizonte, Brazil; ^5^ Neurotec R National Institute of Science and Technology (INCT‐Neurotec R), Faculty of Medicine, Universidade Federal de Minas Gerais (UFMG), Belo Horizonte, Minas Gerais, Brazil; ^6^ Molecular Medicine Postgraduate Program, School of Medicine, Universidade Federal de Minas Gerais (UFMG), Belo Horizonte, Minas Gerais, Brazil; ^7^ Older Adult's Psychiatry and Psychology Extension Program (PROEPSI), School of Medicine, Universidade Federal de Minas Gerais (UFMG), Belo Horizonte, Minas Gerais, Brazil; ^8^ Universidade Federal de Minas Gerais, Belo Horizonte, Minas Gerais, Brazil; ^9^ Cog‐Aging Research Group, Universidade Federal de Minas Gerais (UFMG), Belo Horizonte, Minas Gerais, Brazil; ^10^ Department of Psychiatry, School of Medicine, Federal University of Minas Gerais, Belo Horizonte, Minas Gerais, Brazil; ^11^ Department of Clinical Medicine, Faculty of Medicine, Universidade Federal de Minas Gerais (UFMG), Belo Horizonte, Minas Gerais, Brazil; ^12^ Geriatrics and Gerontology Center Clinical Hospital of Universidade Federal de Minas Gerais, Belo Horizonte, Minas Gerais, Brazil; ^13^ National Institute of Science and Technology Neurotec R (INCT‐MM), Belo Horizonte, Minas Gerais, Brazil

## Abstract

**Background:**

In addition to the acute manifestations of COVID‐19, recent studies have shown that COVID‐19 may be associated with long‐term effects, including cognitive impairment (CI) and functional disability (FI). The aim of this study is to investigate the correlation between cognitive impairment and functional disability in elderly people after the diagnosis of COVID‐19.

**Method:**

Cross‐sectional study nested in a prospective cohort, which recruited elderly people and their respective caregivers after the diagnosis of Covid‐19, confirmed in the laboratory, from 2021 to 2024. All participants signed the Free and Informed Consent Form (CAAE: 31799420.6.0000.5149). Cognition was measured by the Z‐score of cognitive performance on the Dementia Rating Scale and Functionality by the total Functional Independence Measure (FIM) and its subitems. Descriptive statistics, Chi‐square and Spearman correlation were used.

**Result:**

The sample consisted of 80 participants. The mean time between the initial infection and the first evaluation was 10.09 ± 5.97 months (Min.: 2‐Max.: 24). 51.25% of the participants were female, while 57.50% were hospitalized, with a mean age of 73.91 ± 10.20 years; 8.03 ± 3.24 years of schooling, 6.54 ± 3.24 medications in use, Charlson Comorbidity Index of 1.79 ± 1.80 and 3.39 ± 1.63 doses of vaccine for Covid‐19 at the time of diagnosis. 70.87% reported memory difficulty after Covid‐19 and 54.43% of the sample had cognitive disability. The (FIM) indicated the presence of functional disability with a mean of 111.23 ± 11.62. Regarding the diagnosis, 15% had dementia; 62.5% mild cognitive impairment and 22.5% preserved cognition. A correlation was found between the presence of (CC) and (FI) after the diagnosis of COVID‐19 in 92% of the sample (*p* = 0.008). The total (FIM) (rho=‐0.374; *p* = 0.001) and the items related to memory (rho=‐0.374; *p* <0.001), social interaction (rho=‐0.372; *p* = 0.002) and problem‐solving (rho=‐0.366; *p* = 0.002) showed a weak correlation with cognitive impairment. Correlations were also found with the following items: bathing (rho=‐0.248; *p* = 0.038), urine control (rho=‐0.236; *p* = 0.049), transfers (rho=‐265; *p* = 0.026) and gait (rho=‐0.270; *p* = 0.024).

**Conclusion:**

Understanding the relationship between (CC) and (FI) can help in the development of more effective prevention and rehabilitation strategies, contributing to improving the health of the elderly population and their caregivers.

